# Supplementary material for: Distinguishing PEX gene variant severity for mild, severe, and atypical peroxisome biogenesis disorders in Drosophila
Source: bioRxiv. 2024 Nov 19:2024.11.14.623590. Preprint. [Version 2] doi: 10.1101/2024.11.14.623590 (PMC11601393; doi:10.1101/2024.11.14.623590)

Drosophila Pex16 mutants have shortened lifespan, are bang sensitive, and have a climbing defect

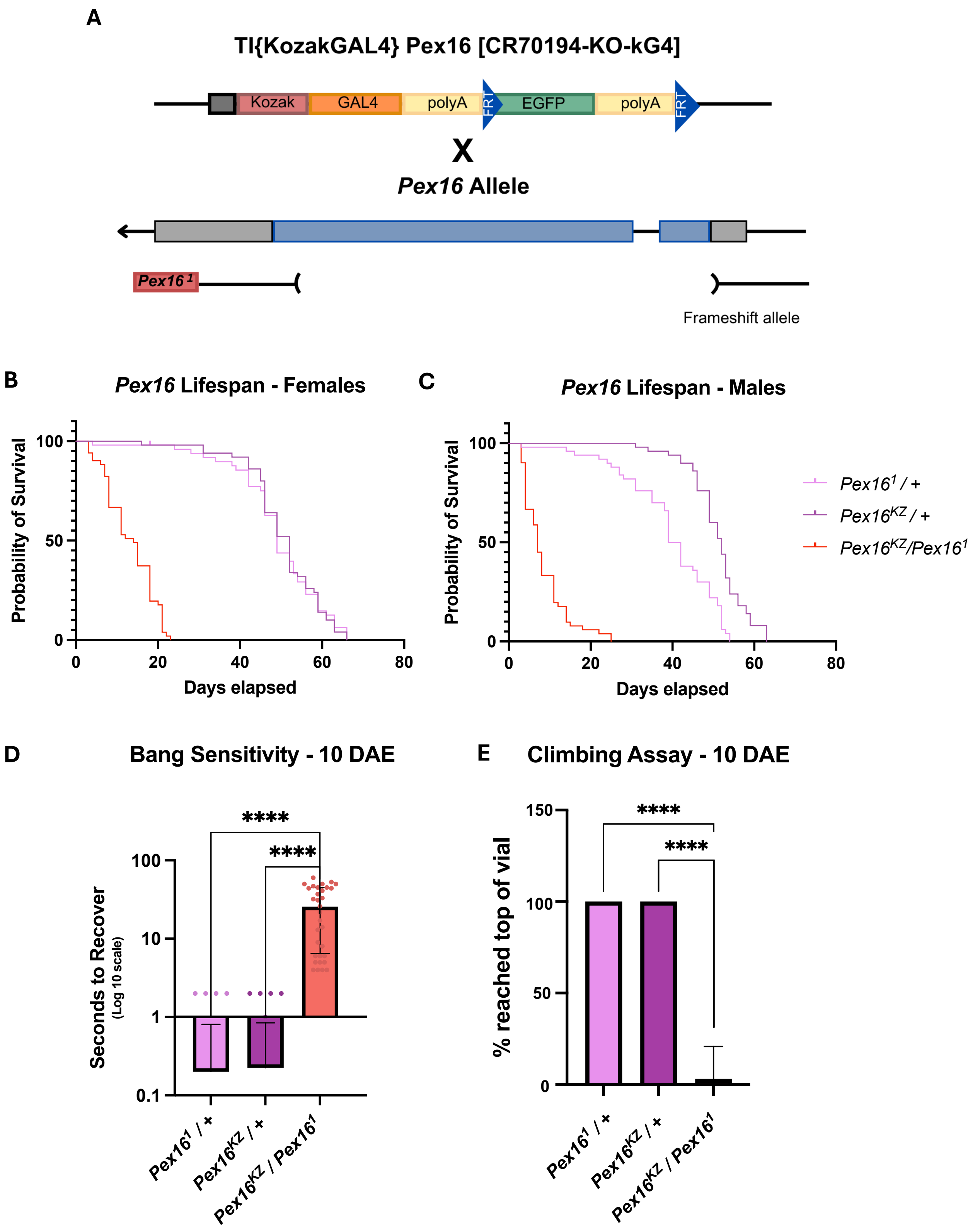

Supplement: Supplement 1 — Figure S1 Drosophila Pex16 mutants have shortened lifespan, are bang-sensitive, and have a climbing defect. (A) Schematic representation of fly Pex16 gene along with one frameshift alleles (Pex161) and Pex16-KozakGAL4 (Pex16KZ). (B) Pex16 female lifespan assay shows that the Pex16 mutants have a shorter lifespan compared to control lines (pink and purple). (C) Pex16 male lifespan assay shows that the Pex16 mutants have a shorter lifespan compared to control lines. D) Pex16 null flies have a significant bangsensitive phenotype (red) compared to controls (pink and purple) observed at 10 days after eclosion (DAE). (E) Pex16 null flies have a significant climbing deficiency (red) compared to controls (pink and purple) observed at 10 days after eclosion. [* = p-value is less than 0.05. ** = p-value is less than 0.01. *** = p-value is less than 0.001. **** = p-value is less than 0.0001] [file media-1.pdf]
