# Supplementary material for: Distinguishing PEX gene variant severity for mild, severe, and atypical peroxisome biogenesis disorders in Drosophila
Source: bioRxiv. 2024 Nov 19:2024.11.14.623590. Preprint. [Version 2] doi: 10.1101/2024.11.14.623590 (PMC11601393; doi:10.1101/2024.11.14.623590)
Supplement: Supplement 2 — Figure S2 Human UAS cDNA PEX16 reference and variant lines. (A) Schematic representation of human PEX16 gene. (B) Schematic representation of human PEX16 protein and variant locations. (C) PEX16 variant table indicates the consequence of the change, pathogenicity prediction, clinical significance, clinical severity in homozygosity and heterozygosity, and conservation in Drosophila. (D) Indicates the observed/expected Mendelian ratio of the F1 generation of human PEX16 variants, in a fly null background. (E) Assessment of the phenotype of indicated genotypes as lethal, viable, or semi-lethal. [file media-2.pdf]

Human UAS cDNA *PEX16* reference and variant lines

A

B

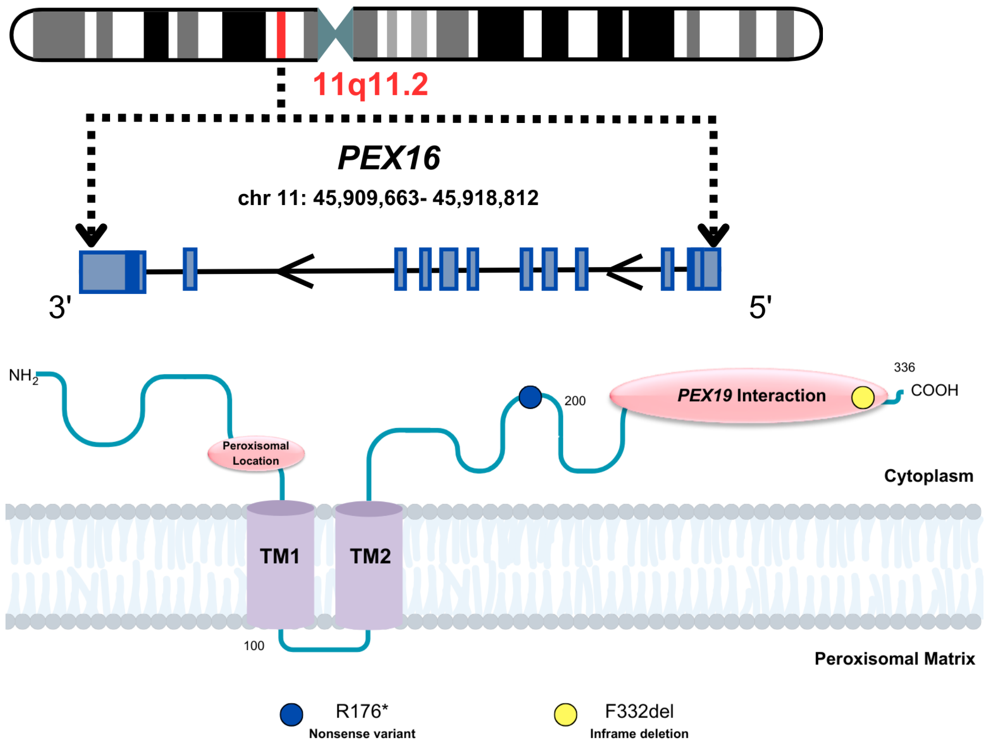

C

PEX16 Variant	Change	Consequence	CADD Score	Clinical Significance	Clinical Severity in Homozygosity	Clinical Severity in Heterozygosity	Conserved in *Drosophila*?
**R176***	NM_004813.4 c.526C.T p.Arg176Ter	Nonsense	37	Pathogenic	**Severe PBD-ZSD**	Not seen	Yes
**F332del**	NM_0048813.4 c.995_997del p.Phe332del	Inframe deletion	-	Likely pathogenic	**Atypical PBD-ZSD**, presenting as ataxia	Not seen	Yes

D

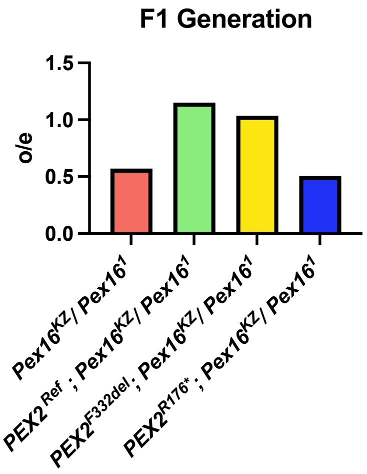

E

Genotype	F1 Progeny
$Pex16^{1}$ / $Pex16^{KZ}$	Semi-lethal
$PEX16^{Ref}$; $Pex16^{1}$ / $Pex16^{KZ}$	Viable
$PEX16^{F332del}$; $Pex16^{1}$ / $Pex16^{KZ}$	Viable
$PEX16^{R176*}$; $Pex16^{1}$ / $Pex16^{KZ}$	Semi-lethal
