# Supplementary figures and images for: Distinguishing PEX gene variant severity for mild, severe, and atypical peroxisome biogenesis disorders in Drosophila
Source: bioRxiv. 2024 Nov 19:2024.11.14.623590. Preprint. [Version 2] doi: 10.1101/2024.11.14.623590 (PMC11601393; doi:10.1101/2024.11.14.623590)

# Pex3 immunostaining in 3<sup>d</sup> instar larva body wall 6 muscle

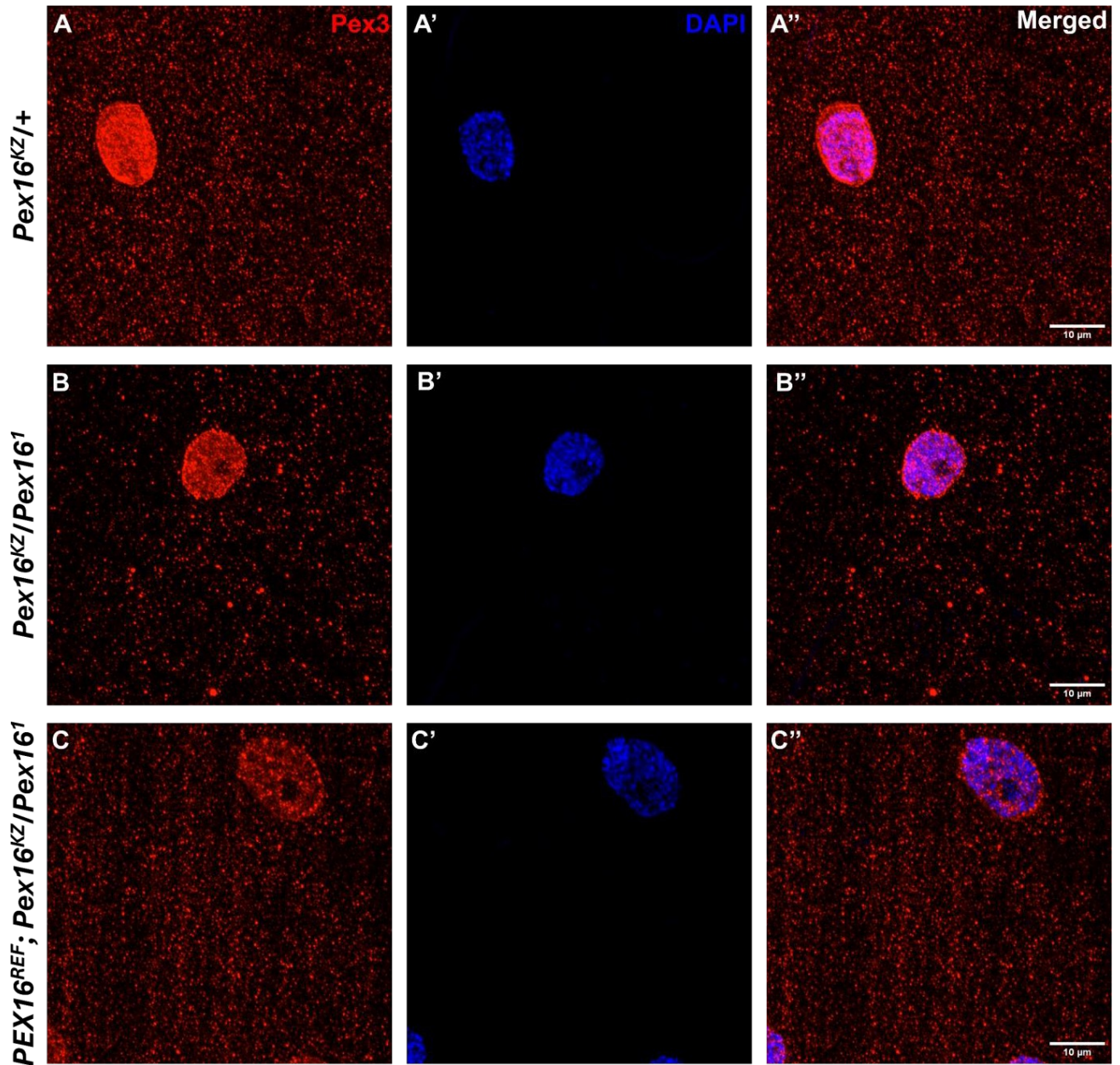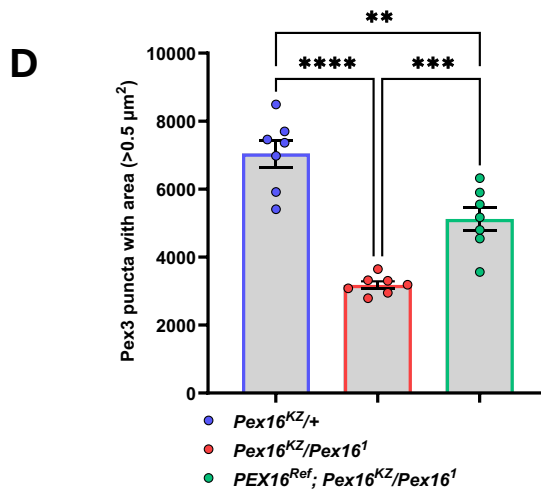

Supplement: Supplement 3 — Figure S3 Human PEX16 expression in Pex16 null background larvae significantly rescues peroxisomes in 3rd instar larva body wall 6 muscle. (A, A’ & A”) represents control group; (B, B’, & B”) represents Pex16 null flies & (C, C’ & C”) represents human rescue group. (D) Quantification of Pex3 positive puncta between the three genotypes. [**p=0.0011; ***p=0.001; ****p<0.0001] [file media-3.pdf]

# Pex3 immunostaining in direct flight muscle (DFM49) in adult flies

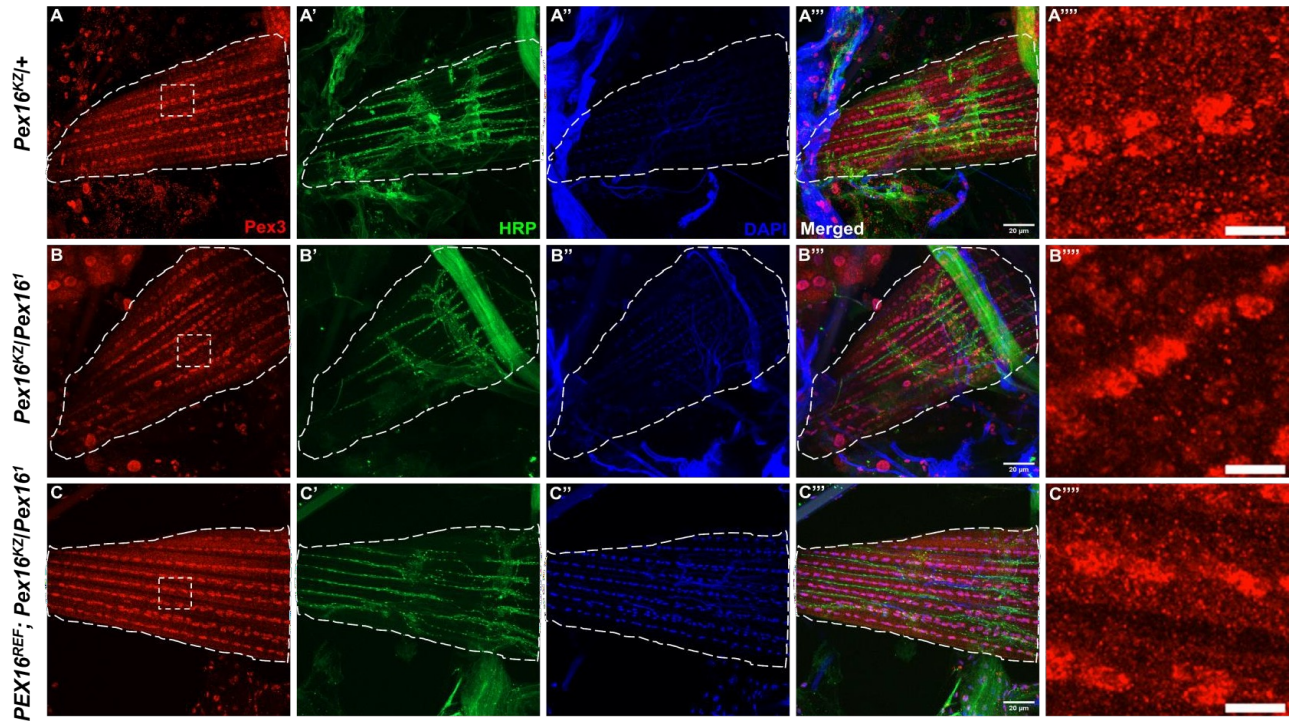

Supplement: Supplement 5 — Figure S5 Human PEX16 expression in Pex16 null background significantly rescues peroxisome number in direct flight muscle (DFM49) in adult flies. (A, A’, A” & A”‘) represents control group; (B, B’, B” & B”‘) represents Pex16 null flies & (C, C’, C” & C”‘) represents rescue group. (A”“, B”“ & C”“) illustrates the closure look at Pex3 puncta within the square box selected ROI for each respective group i.e., control, knockdown & rescue. Scale bar corresponds to 10 μm & 5 μm respectively. DFM49 marked with long dashed line. [file media-5.pdf]
