# Supplementary material for: Distinguishing PEX gene variant severity for mild, severe, and atypical peroxisome biogenesis disorders in Drosophila
Source: bioRxiv. 2024 Nov 19:2024.11.14.623590. Preprint. [Version 2] doi: 10.1101/2024.11.14.623590 (PMC11601393; doi:10.1101/2024.11.14.623590)

Rescue-based humanization of *Pex16*: Behavior assays

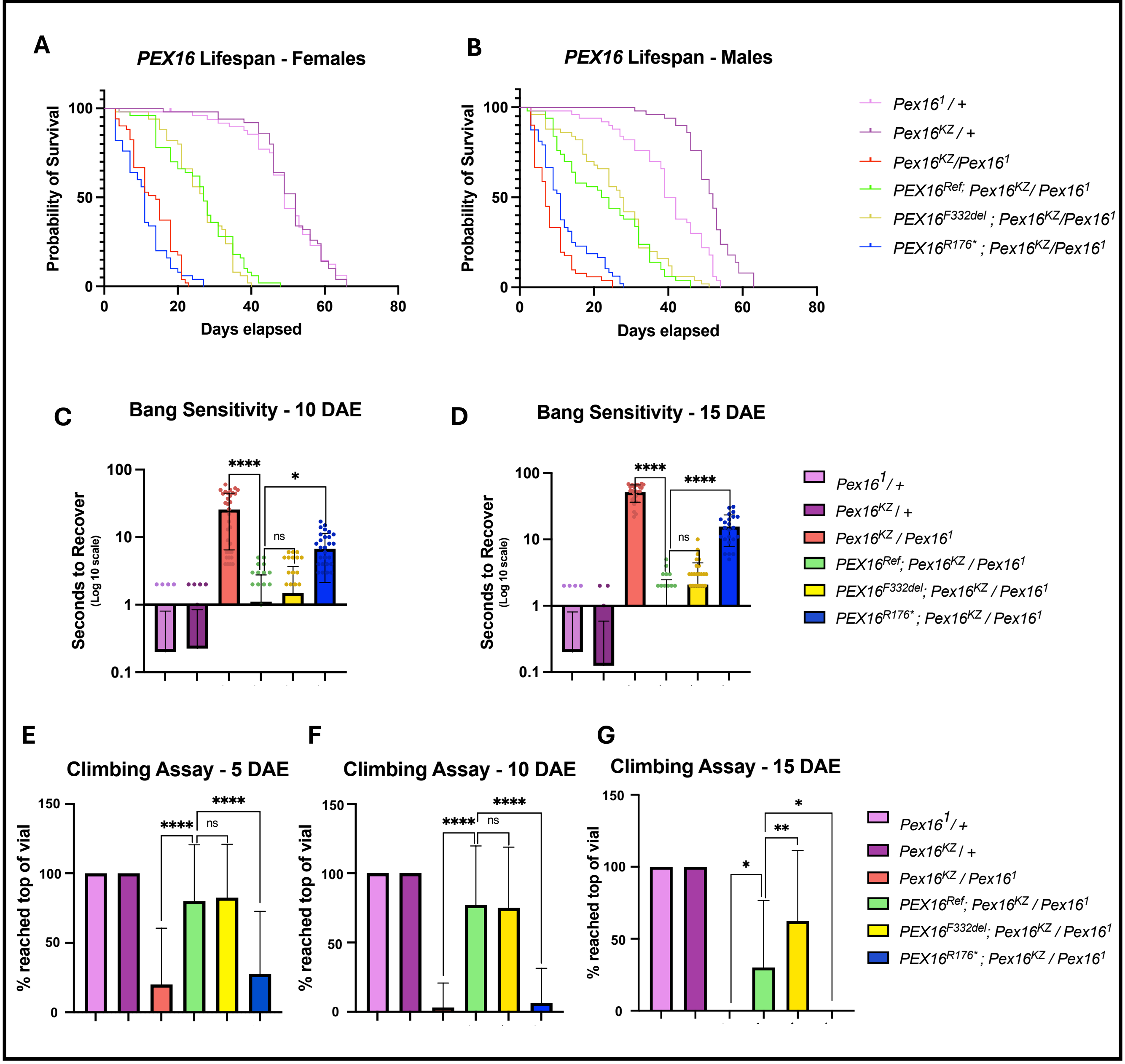

Supplement: Supplement 4 — Figure S4 Rescue-based humanization of Pex16: Behavior Assays. (A) Lifespan analysis of PEX16Ref and Variant female flies, along with our Pex16 null and control lines. (B) Lifespan analysis of PEX16Ref and Variant male flies, along with our Pex16 null and control lines. (C) Bang sensitivity assay of PEX16Ref and Variant female flies, along with our Pex16 null and control lines, at 10 days after eclosion (DAE). (D) Bang sensitivity assay of PEX16Ref and Variant female flies, along with our Pex16 null and control lines, at 15 days after eclosion. (E-G) Climbing assay of PEX16Ref and Variant female flies, along with our Pex16 null and control lines, at 5 days, 10 days, and 15 days after eclosion. [* = p-value is less than 0.05. ** = p-value is less than 0.01. *** = p-value is less than 0.001. **** = p-value is less than 0.0001] [file media-4.pdf]
